# The effects on investor sentiment and business cycles on the systematic risk of an ESG portfolio

**DOI:** 10.1371/journal.pone.0341348

**Published:** 2026-11-05

**Authors:** Edgardo Cayon, Ravi Shukla

**Affiliations:** 1 EGADE Business School, Tecnologico de Monterrey, Mexico City, Mexico; 2 Syracuse University, Whitman School of Management, Syracuse, New York, United States of America; Universitat Jaume I, SPAIN

## Abstract

In this study, we analyze the time varying nature of the systematic risk of an ESG portfolio to common sources of systematic risk. We model the conditional covariances and variances of different sources of systematic risk such as the world stock index, risk aversion as a proxy for investment sentiment (fear), and changes in interest rates. Afterwards, we test for sources of transmission of volatility to ESG systematic risk by controlling for different stages of the business cycle and rising interest rates. Our main finding is that investment sentiment (fear) is not a statistically significant source of systematic risk to the ESG portfolio in the expansion phases of the business cycle but becomes highly relevant in the trough and recession phases and during periods of rising interest rates.

## Introduction

In recent years, the ESG industry has experienced exponential growth. According to a 2022 report by PWC, the assets under management (AUM) via mandates, mutual funds and private markets were estimated to be in the order of 18.4 trillion dollars. The same report estimates that in the asset wealth management industry (AWM) as a whole, ESG investments amounted to 14.4% of the total, and that based on their forecast and in a conservative scenario this amount can rise by the year 2026 to 21.5% of total investments [1]. However, due to changing market conditions, especially those related to tightening monetary policy during the year 2023, Morningstar reported that for the last quarters of 2023 the growth of sustainable funds practically flattened to 0.03% as compared to the general funds market where the growth was estimated at 0.5%. Furthermore, in the first quarter of 2024, sustainable funds worldwide have a net investment of USD 900 million driven mostly by Europe (10.9 billion) while the other two major markets the US and Japan experienced outflows in the order of −8.8 and −1.7 billion, respectively. Finally, the number of new ESG funds has shown a steady decline from 176 new funds at the end of 2023 to just 97 to the first quarter of 2024, marking the lowest level ever for this type of ESG investment since 2021 [2].

This poses an intriguing question regarding whether ESG investing is driven by economic fluctuations connected to the business cycle and changes in monetary policy. The link between portfolio performance and the business cycle has been empirically tested multiple times and the results can be consistent with both rational and irrational models of investor behavior [3]. The motivation for the present paper is derived from the relationship between business conditions and stock markets and its effects of an ESG portfolio after controlling for common factors.

The relationship between business conditions and stock markets has been well documented [4]. found that there was a negative correlation between the expected returns of corporate bonds and stocks and business conditions in the peaks and troughs parts of an economic cycle. This means that during the peak part of the cycle expected returns were low and corporate bonds and stock prices were high, while in the troughs, expected returns were high and corporate bonds and stock prices were low [5]. found that macroeconomic variables such as the spread between long and short interest rates, inflation, industrial production, and the spreads among different corporate bond classes were the principal systematic factors driving the expected returns of stock prices.

In the present paper, our aim is to analyze the impact that the business cycle has on an ESG portfolio from three common factors, namely the world stock market, the US interest rate as a proxy for monetary policy impact, and global risk aversion as a proxy for investor sentiment (fear). One of our main findings was that when we use global risk aversion as a proxy for investor sentiment (fear) there is a statistically significant impact on the discount rate of expected returns of an ESG portfolio with respect to changes in the business cycles.

This paper contributes to the current debate about positive or negative investor preferences and their effect of the discount rate of ESG investments. Recently [6], applying a simple framework developed by [7], argues that the expected returns of highly rated ESG investments are lower than those estimated by a general equilibrium asset pricing model such as the CAPM. The reason driving the disequilibrium is that positive investors sentiment towards ESG causes a lower discount rate due to overbuying that in the short run, tends to drive the price of the stock up. However, as the discount rate adjusts to equilibrium once investor preferences change, the price of the stock goes down and, in the long run, these investments would have a higher discount rate [8]. using a green factor asset pricing model found that the outperformance of environmentally friendly stocks versus traditional ones was positively correlated with investor sentiment toward climate concerns. However, it is important to remember that the proxies for investor sentiment are market data, surveys and text analysis and the question which proxy is preferable is still a matter of current research [9].

From the portfolio performance point of view [10], provide a comprehensive literature review on the subject and conclude that the underperformance of ESG investments can be attributed to non-financial objectives. These non-financial objectives can lead to financial bubbles. From the theoretical point of view [11], propose an equilibrium model in which the non-pecuniary benefits of holding an ESG portfolio explain why investors are willing to accept a lower-than-expected discount rate versus traditional ones [12]. support the role of non-pecuniary benefits in ESG, in a survey conducted among a group of large institutional investors, the authors found that climate risk was considered in the investment decision process from both the financial and non-financial point of view [13]. propose an ESG adjusted CAPM for finding an ESG optimal tangency portfolio and found that the ESG constrained Sharpe ratio was lower than the unconstrained one.

[14] point out the shortfalls of ESG investing and the possible formation of financial bubbles due to lack of market transparency by the rating industry. The problem lies in the lack of regulation and common performance measures across the different types of ESG investments, which in their opinion resembles those of the mortgage-backed securities that triggered the global financial crisis in 2008 [15]. argue that the different metrics used for measuring ESG performance vary enormously among the different suppliers of ESG information leading to a lack of comparability and misinformation [16]. tested this argument by identifying the presence of positive and negative financial bubbles in an array of ESG indices and the google search volume index (GSVI) as a proxy for investor sentiment and found that investor sentiment was the main driver in the formation of both negative and positive bubbles. Another argument for the formation of bubbles, is that ESG investments are prone to manipulation and in some cases have become the perfect vehicle for “greenwashing” given that an ESG label can generate positive investor sentiment and herding behavior, which in turn can lead to overvaluations due to misleading information. Indeed, some argue that ESG investing is just investing and that the ESG label is just an additional factor among many factors for valuing financial performance [17,18]. This line of arguments can be a plausible explanation as to why there is a vast amount of conflicting empirical evidence concerning the financial performance of ESG investments from an asset pricing point of view.

In the case of ESG investments underperformance [19], using a dataset that contained firms that were publicly recognized as good corporate citizens, failed to obtain positive alpha during a three-year period after the announcement [20]. used a four-factor model and ESG rating as an additional factor and their results concluded that there was no evidence that ESG investing led to superior abnormal returns [21]. found that even though high sustainability ranked U.S. mutual funds experienced the largest amount of inflow than those with low rankings, there was no evidence that one type of fund outperformed the other in term of returns [22]. using a discounted cash flow model, found evidence that ESG information was transmitted through both the systematic and idiosyncratic risk channels. In this paper, we add to the literature by analyzing and quantifying the time-varying nature of systematic risk that originates from three common factors, namely the world stock market, the US interest rate as a proxy for monetary policy and global risk aversion as a proxy for investor sentiment (fear).

On the other hand, in the case of overperformance [23], argue that screening for investments using a weighted average Sharpe ratio that accounts for risk adjusted ESG scores provided acceptable performance for a set of regional and global portfolios [24]. provide evidence that for the firms that compose the S&P 500, ESG ratings were positively correlated with sales for the larger firms and with capitalization for the smaller firms [25]. using the bid-ask spread as a proxy for market information asymmetry, found that ESG disclosures increased market efficiency due to smaller bid-ask spreads [26]. used ESG ratings to demonstrate that it is possible to construct a hedge mimicking portfolio that can mitigate climate risk.

At the international level, the evidence is also contradicting [27]. provided evidence that in the case of China a portfolio composed of companies with low dispersion ESG ratings consistently outperformed those with high dispersion of ESG ratings [28]. provide evidence, using a standard test, that in the biggest economies of continental Europe (namely Germany and France), there is evidence of herding behavior toward ESG investments [29]. provided evidence that in the case of Japan the impact of ESG ratings in financial performance was similar to proxy measures of market sentiments [30]. provides evidence that in the case of Korea, different markets data-based proxies for market sentiment are consistent in explaining the variation of market returns when the level of market uncertainty is high [31]. in the case of England, analyzed the performance of value weighted ESG portfolio versus traditional ones between 2004 and 2011 and found that the ESG portfolios were more resilient to economic crisis and adverse economic conditions [32]. using cointegration analysis found that in the case of India there was no significant causality between investor sentiment and ESG investments. In the paper, we propose the use of a proxy for a world market index to model the systematic risk derived from this common factor to an ESG portfolio. The remainder of the paper is as follows, in section 2 we discuss our data and choice of variables; in section 3 we discuss our methods, in section 4 we discuss the results and in section 5 we conclude.

## Data and methods

### Data

The proxy for the ESG portfolio is the daily closing price of the S&P 500 ESG index (ticker: SPXESUP) which is a market weighted capitalization index of companies that outperform their peers in sustainability criteria. We use 3 common factors that transmit financial shocks to the above-mentioned portfolio. In an asset pricing framework, the first factor is the World Index (ticker: WORLD), which is our proxy for the impact of the global market movements on the ESG portfolio and is a market weighted index that covers 85% of the world market capitalization. The second factor is the CBOE volatility index (ticker: VIX) which is our proxy for the impact of investor sentiment (fear) and is an index that measures the implied constant volatility for the next 30 days of the US stock market [33,34] conducts a comprehensive literature review on the effectiveness of the VIX as a proxy for investor sentiment (fear) given its properties as an effective measure of risk aversion. The principal argument is that put buyers, who act as portfolio insurers, are the predominant participants in the options markets. Consequently, an increase in implied volatility is attributed to heightened demand for put options or market expectations of declining prices. This increase in the VIX volatility can be interpreted as an increase in risk aversion or in other words “investor fear.” Finally, the third factor is the Bloomberg Treasury Index (ticker: LUATTRU) which is our proxy for the impact of interest rates on the ESG portfolio. It is an index of US dollar denominated nominal fixed-rated debt issued by the US government with a remaining maturity of 1 year and it excludes strips and short-term treasury bills. This is important because this index is designed to capture changes in market interest rates and reduce jumps in short-term volatility, in other words, the index reflects the immediate impact of interest rates in the market. The frequency of the data for the indices is daily and it spans from the 3^rd^ of May of 2010 to the 6^th^ of May of 2024. Summary statistics for the data are provided in Table 1.

**Table 1:** Descriptive statistics: Daily returns of the ESG portfolio and common factors.

	ESG PORTFOLIO	WORLD INDEX	VIX ETN	BLOOMBERG TREASURY INDEX
Mean	0.000411	0.000251	−0.000106	6.19E-05
Median	0.000345	0.000549	−0.003608	8.60E-06
Maximum	0.091458	0.078863	0.768245	0.016679
Minimum	−0.127693	−0.098227	−0.350588	−0.018800
Std. Dev.	0.010807	0.009077	0.075441	0.002886
Skewness	−0.694537	−0.919041	1.136983	−0.004128
Kurtosis	17.08016	15.93600	9.731282	6.554110
Jarque-Bera	30485.80	25999.03	7687.848	1923.712
Probability	0.000000	0.000000	0.000000	0.000000
Sum	1.500505	0.917488	−0.387062	0.226069
Sum Sq. Dev.	0.426764	0.301078	20.79593	0.030432
Observations	3655	3655	3655	3655

Notes: The table shows summary statistics for daily percentage changes for the variables under study in the case of VIX, we used the tradable ETN to capture a proxy of daily price returns, all the data was extracted from Bloomberg.

Additionally, the dates for the dummy variable of periods of rising interest rates are extracted from the Forbes webpage [35] and the dummies for the business cycles from the National Bureau of Economic Research webpage [36] which are used to model the impact of changes in monetary policy and economic conditions on investor sentiment, The dates for the business cycles are shown in Table 2.

**Table 2:** Dates for the business cycles for the period May 4^th^, 2010, to May 6^th^, 2024.

*Business cycle*	*Begins*	*Ends*
Expansion/Peak	May 4^th^, 2010	February 28^th^,2020
Trough/Recession	March 2^nd^, 2020	May 1^st^, 2020
Recession/Expansion	May 4, 2020	May 6^th^, 2024

Source: [36] and the dates can be found at https://www,nber,org/research/business-cycle-dating and https://www.nber.org/research/data/us-business-cycle-expansions-and-contractions.

Therefore, we modeled three dummies for each business cycle where the dates of the business cycle take the value of 1 and 0 otherwise for dates that are outside the respective business cycle, In the case of the rising interest rates, the dummy takes the value of 1 in the period where the fed keeps rising the interest rate until the next cut, for example, the first period of rising interest rate in our sample went from December 17^th^, 2015 until September 30^th^, 2019, in this case the period would take the value of 1, from August 1^st^, 2019 to March 16^th^, 2022 there was a period of cuts by the federal reserve and therefore the value of the dummy would be zero, from March 22^nd^, 2022 to the end of the sample is a period of rising interest rates therefore the value of the dummy is 1, all other dates in the sample take the value of zero. The purpose of this variable is to capture the impact that tighter monetary policy conditions have on investor sentiment towards ESG. Also, for robustness purposes and to examine the effects of changes in volatility regimes, we constructed a dummy variable that takes the value of 1 when the VIX is above the accepted threshold for high volatility and 0 otherwise. We used the threshold value of 23.81, following [37] who found that this is the cut-off point at which most active VIX futures traders, both short and long, change their investment strategies. Therefore, when the VIX index is above 23.81, it is considered a high-volatility regime; when it is below this threshold, it is considered a low-volatility regime.

## Method

We begin by modelling the time-varying volatility interaction of the S&P ESG index returns (ESG), the Bloomberg world index (WI), the VIX volatility index (VX), and the Bloomberg Treasury index (TR), The first step is to model a set of different autoregressive (AR) process to obtain a residual series that represent the idiosyncratic risk of each variable (Equation 1):


$$\begin{array}{c} r_{ESG,t}=\alpha_{\text{0}}+\phi_{\text{1}}r_{ESG,t-1}+\phi_{\text{2}}r_{ESG,t-2}+\phi_{\text{3}}r_{WI,t}+\phi_{\text{4}}r_{VX,t}+\phi_{\text{5}}r_{TR,t}+\varepsilon_{ESG,t} \end{array}$$

$$\begin{array}{c} r_{WI,t}=\alpha_{\text{0}}+\phi_{\text{1}}r_{WI,t-1}+\phi_{\text{3}}r_{ESG,t}+\phi_{\text{4}}r_{VX,t}+\phi_{\text{5}}r_{TR,t}+\varepsilon_{WI,t} \end{array}$$

$$\begin{array}{c} r_{VX,t}=\alpha_{\text{0}}+\phi_{\text{1}}r_{VX,t-1}+\phi_{\text{3}}r_{ESG,t}+\phi_{\text{4}}r_{WI,t}+\phi_{\text{5}}r_{TR,t}+\varepsilon_{VX,t} \end{array}$$

$$\begin{array}{c} r_{TR,t}=\alpha_{\text{0}}+\phi_{\text{1}}r_{TR,t-1}+\phi_{\text{3}}r_{ESG,t}+\phi_{\text{4}}r_{WI,t}\phi_{\text{5}}r_{VX,t}+\varepsilon_{TR,t} \end{array}$$ (1)

where $r_{ESG,t}$, $r_{WI,t}$, $r_{VX,t}$ and $r_{TR,t}$ are the daily returns of the S&P ESG index, Bloomberg world index, VIX volatility index, and treasury index, respectively, and $\varepsilon_{ESG,t}$, $\varepsilon_{WI,t}$, $\varepsilon_{VX,t}$ and $\varepsilon_{TR,t}$ are the residuals obtained for each of the variables from the AR processes in equation (1). In order to choose the optimal number of lags in equation (1), we took the best fit in the correlogram of residuals for each variable, which was one lag for the WI, VX and TR variables, and in the specific case of the ESG there were two lags.

We use the residuals to model a multivariate GARCH (M-GARCH) with a diagonal BEKK specification with a full rank matrix to ensure that the coefficient matrices are positive definite using the following specification (equation 2):


$$\begin{array}{c} \begin{array}{c} h_{ESG_{t}}=\alpha_{\text{0}}+\alpha_{1,ESG_{t}}{\varepsilon^{\text{2}}}_{ESG_{t-1}}+\alpha_{2,ESG_{t}}h_{ESG_{t-1}}\\ h_{WI_{t}}=\alpha_{\text{0}}+\alpha_{1,WI_{t}}{\varepsilon^{\text{2}}}_{WI_{t-1}}+\alpha_{2,WI_{t}}h_{WI_{t-1}}\\ h_{VX_{t}}=\alpha_{\text{0}}+\alpha_{1,VX_{t}}{\varepsilon^{\text{2}}}_{VX_{t-1}}+\alpha_{2,VX_{t}}h_{VX_{t-1}}\\ h_{TR_{t}}=\alpha_{\text{0}}+\alpha_{1,TR_{t}}{\varepsilon^{\text{2}}}_{TR_{t-1}}+\alpha_{2,TR_{t}}h_{TR_{t-1}}\\ h_{WI_{t},ESG_{t}}=\alpha_{\text{0}}+\alpha_{1,WI_{t}ESG_{t}}\varepsilon_{WI_{t-1}}\varepsilon_{ESG_{t-1}}+\alpha_{2,WI_{t}ESG_{t}}h_{WI_{t-1},ESG_{t-1}}\\ h_{WI_{t},VX_{t}}=\alpha_{\text{0}}+\alpha_{1,WI_{t}VX_{t}}\varepsilon_{WI_{t-1}}\varepsilon_{VX_{t-1}}+\alpha_{2,WI_{t}VX_{t}}h_{WI_{t-1},VX_{t-1}}\\ h_{WI_{t},TR_{t}}=\alpha_{\text{0}}+\alpha_{1,WI_{t}TR_{t}}\varepsilon_{WI_{t-1}}\varepsilon_{TR_{t-1}}+\alpha_{2,WI_{t}TR_{t}}h_{WI_{t-1},TR_{t-1}}\\ h_{VX_{t},ESG_{t}}=\alpha_{\text{0}}+\alpha_{1,VX_{t}ESG_{t}}\varepsilon_{VX_{t-1}}\varepsilon_{ESG_{t-1}}+\alpha_{2,VX_{t}ESG_{t}}h_{VX_{t-1},ESG_{t-1}}\\ h_{VX_{t},TR_{t}}=\alpha_{\text{0}}+\alpha_{1,VX_{t}TR_{t}}\varepsilon_{VX_{t-1}}\varepsilon_{TR_{t-1}}+\alpha_{2,VX_{t}TR_{t}}h_{VX_{t-1},TR_{t-1}}\\ h_{TR_{t},ESG_{t}}=\alpha_{\text{0}}+\alpha_{1,TR_{t}ESG_{t}}\varepsilon_{TR_{t-1}}\varepsilon_{ESG_{t-1}}+\alpha_{2,TR_{t}ESG_{t}}h_{TR_{t-1},ESG_{t-1}}\\ \left[\begin{array}{c} @l@\varepsilon_{ESG_{t}}\\ \varepsilon_{WI_{t}}\\ \varepsilon_{VX_{t}}\\ \varepsilon_{TR_{t}} \end{array}\right]|\Omega_{t-1}\sim \;N\left(\left[\begin{array}{c} @l@0\\ 0\\ 0\\ 0 \end{array}\right],\left[\begin{array}{c} @l@h_{ESG_{t}}h_{ESG_{t},WI_{t}}h_{ESG_{t},VX_{t}}h_{ESG_{t},TR_{t}}\\ h_{WI_{t},ESG_{t},}h_{WI_{t}}h_{WI_{t},VX_{t}}h_{WI_{t},TR_{t}}\\ h_{VX_{t},ESG_{t}}h_{VX,WI_{t}}h_{VX_{t}}h_{VX_{t},TR_{t}}\\ h_{TR_{t},ESG_{t}}h_{TR_{t},WI_{t}}h_{TR_{t},VX_{t}}h_{TR_{t}} \end{array}\right]\right) \end{array} \end{array}$$ (2)

where $h_{{TR}_{t}}$, $h_{{VX}_{t}}$, $h_{{WI}_{t}}$, and $h_{{ESG}_{t}}$ are the conditional variance of filtered returns for the Bloomberg Treasury Index (TR), VIX volatility Index (VX), World Index (WI) and S&P ESG index (ESG), respectively. $h_{{TR}_{t},\;{VX}_{t}}$, $h_{{TR}_{t},\;{WI}_{t}}$, $h_{{TR}_{t},\;{ESG}_{t}}$, $h_{{VX}_{t},\;{WI}_{t}}$, $h_{{VX}_{t},\;{ESG}_{t}}$, $h_{{WI}_{t},\;{ESG}_{t}}$ are the conditional covariances between the various index pairs.

Using the fitted values from the conditional variances and covariances from the M-GARCH model, we can use equation (3), in which we decompose the betas in terms of variances and covariances to capture the interactions among the different factors. $\beta_{WI_{t}}$ is defined in terms of the other two betas for simplifications purposes. By allowing for this specification, we can compute the *β*s of a three-factor model in which the Bloomberg Treasury Index (TR), the VIX Volatility index (VX), and the World Index (WI) explain the variance of the S&P ESG index (ESG):


$$\begin{array}{c} \begin{array}{c} \beta_{TR_{t}}=\frac{\left(h_{WI_{t}}h_{TR_{t},VIX_{t}}\right)-\left(h_{TR_{t},WI_{t}}h_{VX_{t},WI_{t}}\right)\left(h_{VX_{t},WI_{t}}h_{TR_{t},ESG_{t}}\right)-\left(h_{TR_{t},WI_{t}}h_{VX_{t},WI_{t}}\right)-\left(h_{VX_{t},WI_{t}}h_{TR_{t},VX_{t}}\right)-\left(h_{TR_{t},WI_{t}}h_{VX_{t}}\right)-\left(h_{WI_{t}}h_{TR_{t},ESG_{t}}\right)-\left(h_{TR_{t},WI_{t}}h_{WI_{t},ESG_{t}}\right)}{\left(h_{WI_{t}}h_{TR_{t},VIX_{t}}\right)-\left(h_{TR_{t},WI_{t}}h_{VX_{t},WI_{t}}\right)\left(h_{VX_{t},WI_{t}}h_{TR_{t}}\right)-\left(h_{TR_{t},WI_{t}}h_{TR_{t},VX_{t}}\right)-\left(h_{VX_{t},WI_{t}}h_{TR_{t},VX_{t}}\right)-\left(h_{TR_{t},WI_{t}}h_{VX_{t}}\right)\left(h_{WI_{t}}h_{TR_{t}}\right)-\left(h_{TR_{t},WI_{t}}h_{TR_{t},WI_{t}}\right)}\\ \beta_{VX_{t}}=\frac{\left(h_{WI_{t}}h_{TR_{t}}\right)-\left(h_{TR_{t},WI_{t}}h_{TR_{t},WI_{t}}\right)\left(h_{VX_{t},WI_{t}}h_{TR_{t},ESG_{t}}\right)-\left(h_{TR_{t},WI_{t}}h_{VX_{t},WI_{t}}\right)-\left(h_{VX_{t},WI_{t}}h_{TR_{t}}\right)-\left(h_{TR_{t},WI_{t}}h_{TR_{t},VX_{t}}\right)\left(h_{WI_{t}}h_{TR_{t},ESG_{t}}\right)-\left(h_{TR_{t},WI_{t}}h_{WI_{t},ESG_{t}}\right)}{\left(h_{WI_{t}}h_{TR_{t}}\right)-\left(h_{TR_{t},WI_{t}}h_{TR_{t},WI_{t}}\right)\left(h_{VX_{t},WI_{t}}h_{TR_{t},VX_{t}}\right)-\left(h_{TR_{t},WI_{t}}h_{VX_{t}}\right)-\left(h_{VX_{t},WI_{t}}h_{TR_{t}}\right)-\left(h_{TR_{t},WI_{t}}h_{TR_{t},VX_{t}}\right)\left(h_{WI_{t}}h_{TR_{t},VIX_{t}}\right)-\left(h_{TR_{t},WI_{t}}h_{VX_{t},WI_{t}}\right)}\\ \beta_{WI_{t}}=\frac{h_{TR_{t},ESG_{t}}-\beta_{TR_{t}}h_{TR_{t}}-\beta_{VX_{t}}h_{TR_{t},VX_{t}}}{h_{TR_{t}WI_{t}}} \end{array} \end{array}$$ (3)

The advantage of equation (3) under an asset pricing context (for the theoretical explanation of how the beta is decomposed into systematic and idiosyncratic risk please refer to [38]) is that we can decompose the variance of S&P ESG index (ESG) into systematic and idiosyncratic risk components using the specification in equation (4):


$$\begin{array}{c} h_{ESG_{t}}={\beta_{TR_{t}}}^{\text{2}}h_{TR_{t}}+{\beta_{VX_{t}}}^{\text{2}}h_{VX_{t}}+{\beta_{WI_{t}}}^{\text{2}}h_{WI_{t}}+2\beta_{TR_{t}}\beta_{VX_{t}}h_{TR_{t},VX_{t}}+2\beta_{TR_{t}}\beta_{WI_{t}}h_{TR_{t},WI_{t}}+2\beta_{VX_{t}}\beta_{WI_{t}}h_{VX_{t},WI_{t}}+h_{\varepsilon_{t}} \end{array}$$ (4)

where $h_{ESG_{t}}$ is the variance of the S&P ESG index (ESG), ${\beta_{TR_{t}}}^{\text{2}}h_{TR_{t}}$, ${\beta_{VX_{t}}}^{\text{2}}h_{VX_{t}}$, and ${\beta_{WI_{t}}}^{\text{2}}h_{WI_{t}}$ are the parts of the variance attributed to the systematic shocks transmitted by the Bloomberg Treasury Index (TR), the VIX Volatility index (VX), and the World Index (WI), respectively. The terms $2\beta_{TR_{t}}\beta_{VX_{t}}h_{TR_{t},VX_{t}}$, $2\beta_{TR_{t}}\beta_{WI_{t}}h_{TR_{t},WI_{t}}$, and $2\beta_{TR_{t}}\beta_{WI_{t}}h_{TR_{t},WI_{t}}$ account for the effect of the covariance between the various indexes. Finally, $h_{\varepsilon_{t}}$ is the part of the variance attributed to idiosyncratic factors or unique risk. Therefore, we can decompose the variance of the S&P ESG index (ESG) into its systematic and idiosyncratic risk components using equation (5):


$$\begin{array}{c} \begin{array}{c} u_{SYS,ESG_{t}}=\frac{{\beta_{TR_{t}}}^{\text{2}}h_{TR_{t}}+{\beta_{VX_{t}}}^{\text{2}}h_{VX_{t}}+{\beta_{WI_{t}}}^{\text{2}}h_{WI_{t}}+2\beta_{TR_{t}}\beta_{VX_{t}}h_{TR_{t},VX_{t}}+2\beta_{TR_{t}}\beta_{WI_{t}}h_{TR_{t},WI_{t}}+2\beta_{VX_{t}}\beta_{WI_{t}}h_{VX_{t},WI_{t}}}{h_{ESG_{t}}}\\ u_{IDIO,ESG_{t}}=\frac{h_{\varepsilon_{t}}}{h_{ESG_{t}}} \end{array} \end{array}$$ (5)

Once we obtain the time series for the percentage of variance of the S&P ESG index (ESG) explained by systematic risk from equation (5), we can proceed to test if there is a statistically significant difference between the different sources of systematic risk (represented by the conditional covariances from equation (2)) among different busyness cycles as dated by the national bureau of economic research and allowing for the interaction of periods with rising interest rates. For this purpose, we regress the different sources of systematic risk on a constant, a lagged value to control for serial correlation and dummy variables for the different business cycles and periods of rising interest rates following the specification in equation (6):


$$\begin{array}{c} u_{\text{SYS,\textbackslash{} SOURCE},\;t}=\varphi_{\text{0}}+\varphi_{\text{1}}u_{\text{SYS,\textbackslash{} SOURCE},\;t-1}+\varphi_{\text{2}}I_{\text{exp\textbackslash{} peak},\;t}+\varphi_{\text{3}}I_{\text{trough\textbackslash{} rec},\;t}+\varphi_{\text{4}}I_{rir,t}+\varphi_{\text{5}}I_{hv,t}+e_{i,t} \end{array}$$

$$\begin{array}{c} u_{\text{SYS,\textbackslash{} SOURCE},\;t}=\varphi_{\text{0}}+\varphi_{\text{1}}u_{\text{SYS,\textbackslash{} SOURCE},\;t-1}+\varphi_{\text{2}}I_{\text{trough\textbackslash{} rec},\;t}+\varphi_{\text{3}}I_{\text{rec\textbackslash{} peak},\;t}+\varphi_{\text{4}}I_{rir,t}+\varphi_{\text{5}}I_{hv,t}+e_{i,t} \end{array}$$ (6)

Where $u_{\text{SYS,\textbackslash{} SOURCE},\;t}$ is the portion of % systematic risk contributed by the different sources of conditional variance and covariance as a % of total systematic variance (for example in the specific case of the Bloomberg Treasury Index the contribution % of systematic risk is defined as $u_{\text{SYS,TR},\;t}=\frac{\beta_{\text{TR},\;t}^{\text{2}}h_{\text{TR},\;t}}{h_{\text{ESG},\;t}}$, and $I_{j,t}$ is the indicator (dummy) variable that takes the value of one for the respective dates of the different business cycles (expansion to peak, trough to recession, and recession to peak), the dates where the reference interest rate set by the FED is in a rising cycle and the high volatility regime is explained in section 2.

## Results and discussion

In Fig 1, we can observe the dynamic path of $u_{\text{SYS},\text{ESG},t}$ and $u_{\text{IDIO},\text{ESG},t}$ during the period under observation, the graph shows the decomposition of the conditional volatility of our proxy for the ESG portfolio (S&P ESG index) into systematic volatility ($u_{\text{SYS},\text{ESG},t}$) sourced by the World Index (WI), Bloomberg Treasury Index (TR), and the CBOE volatility index (VX) and their respective covariances derived from equation (4), from the figure there is peak in unique risk or idiosyncratic volatility ($u_{\text{IDIO},\text{ESG},t}$) in March 2022, which mark the beginning of our last period of rising interest rates.

**Fig 1:**
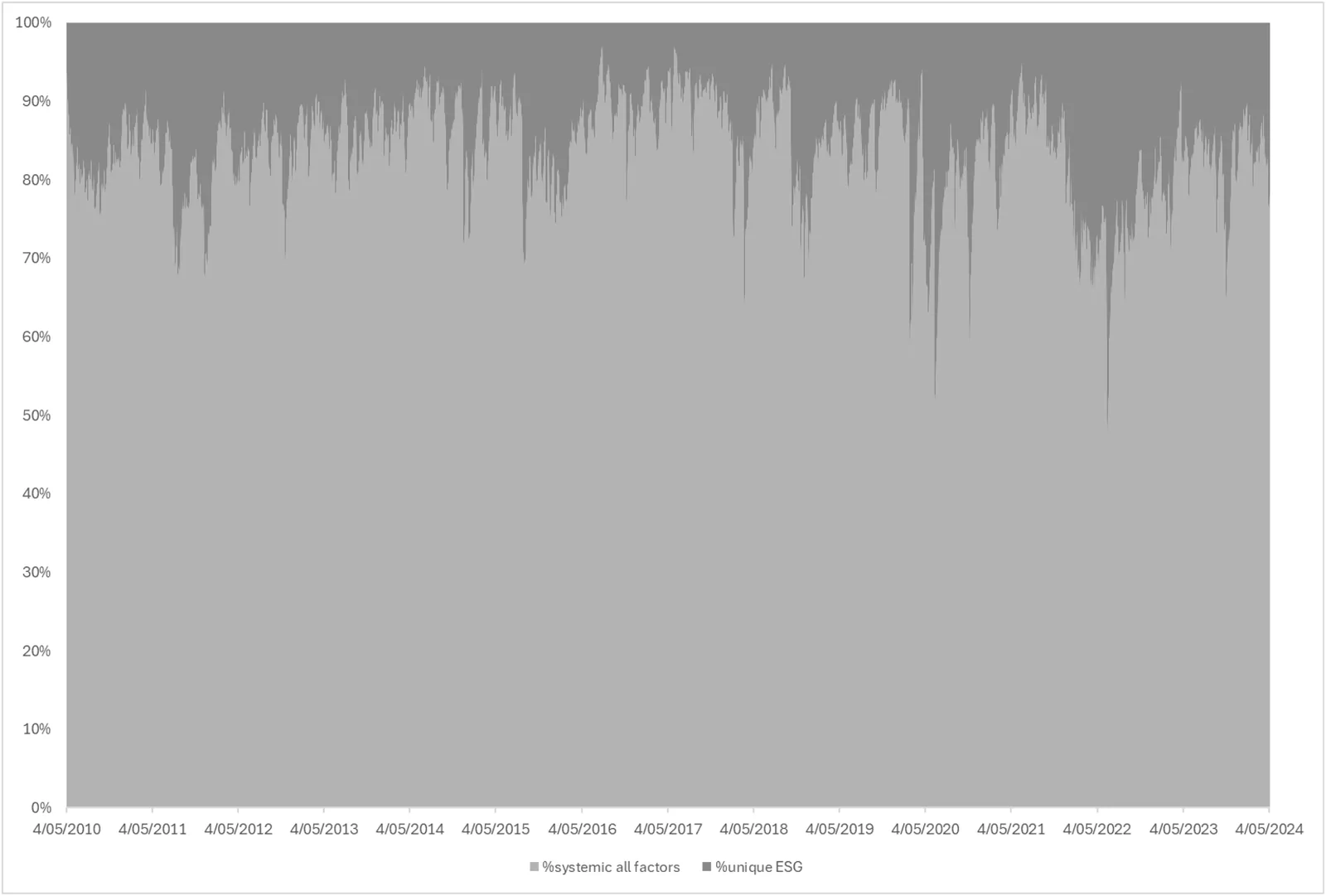
Conditional variance decomposition. ESG portfolio return from May 4^th^. **2010.** to **May 24**^th^**. 2024.** Note: This Figure plots proportion of conditional volatility of ESG portfolio due to systematic volatility shocks from the World Index. VIX and the Bloomberg Treasury Index.

In Table 3, there are the contributions of each factor to total systematic risk expressed as a percentage during different stages of the business cycle, the world index is the factor that contributes most to total systematic risk, followed by our proxy of investment sentiment. The effect of the treasury index and the different covariances among factors is negligible. Interestingly, in the trough/recession stage of the business cycle as expected investor sentiment becomes the major source of total systematic risk. Even when we control for a high volatility regime, it is only during the trough/recession period that investor sentiment (fear), proxied by the VIX, becomes the major source of systematic risk. Therefore, we use the specification in equation (6) to test if there are shocks to systematic risk due to changes of the business cycle, rising interest rates, or high volatility and if these shocks are statistically significant or not.

**Table 3:** Total systematic risk of the ESG portfolio expressed as a percentage contribution of selected factors.

*Period*	$u_{SYS,ESG,t}$	$u_{SYS,WI,t}$	$u_{SYS,VX,t}$	$u_{SYS,TR,t}$	$u_{SYS,}h_{VX_{t},WI_{t}}$	$u_{SYS,}h_{WI_{t},TR_{t}}$	$u_{SYS,}h_{VX_{t},TR_{t}}$
Expansion/Peak	85.47%	77.38%	10.15%	0.61%	−1.00%	−1.37%	−0.30%
Trough/Recession	77.95%	39.64%	45.94%	0.38%	−6.13%	−1.60%	−0.28%
Recession/Expansion	79.98%	65.44%	13.91%	2.63%	0.62%	−2.29%	−0.34%
Rising interest rates	83.11%	69.92%	13.92%	1.70%	−0.22%	−1.81%	−0.38%
High Volatility	75.97%	61.99%	15.61%	1.46%	−1.62%	−1.57%	0.10%

Note: The percentages shown in the table are the conditional variances from the M-GARCH specification of equation (2) using the filtered returns from equation (1). Then we proceed to calculate the time varying betas using the specification in equation (3). The conditional variance of the ESG portfolio is obtained using equation (4) and finally the percentage terms are obtained using equation (5).

There are some interesting results when we test the effect that changes in business cycles and rising interest rates have on the ESG portfolio’s total systematic risk and each of its components. First, total systematic risk ($u_{\text{SYS},\text{ESG},t}$) as calculated in equation (5) allows for the interaction of the factors as independent sources of systematic risk and well as their respective covariances. From Table 4, we observe that there is a statistically significant transmission shock when the business cycle is in the stage of expansion to peak ($\varphi_{2\text{exp\textbackslash{} peak},\;t}$). There is also a statistically significant transmission shock in times of rising interest rates. ($\varphi_{4\text{rir},\;t}$). In both cases, we can observe that the coefficients are positive, which means that that particular stage of the business cycles as well as in the period of rising interest rates systematic risk increases. From the table, we can observe that the stage of trough recession ($\varphi_{3\text{trough\textbackslash{} rec},\;t}$) does not affect total systematic risk. The result obtained for trough recession is different when we allow for transmission shocks from trough to recession and recession to peak (Table 5). From Table 5 we observe that in the stage of recession to peak ($\varphi_{3\text{rec\textbackslash{} peak},\;t}$) the coefficient is significant and negative which can be interpreted that in this stage of the business cycle, the idiosyncratic or unique risk of the ESG portfolio increases as systematic risk decreases. Finally, when volatility increases ($\varphi_{5hv,t}$), we observe a similar effect in which the unique risk of the ESG portfolio increases as systematic risk decreases.

**Table 4:** Tests of transmission of shocks from different stages of the business cycle, rising interest rates and high volatility to different sources of systematic risk of the ESG portfolio (expansion to peak to trough and recession).

**Dependent variable:** $u_{SYS,SOURCE,t}$	$u_{SYS,ESG,t}$	$u_{SYS,WI,t}$	$u_{SYS,VX,t}$	$u_{SYS,TR,t}$	$u_{SYS,}h_{VX_{t},WI_{t}}$	$u_{SYS,}h_{WI_{t},TR_{t}}$	$u_{SYS,}h_{VX_{t},TR_{t}}$
**Coefficient**							
$\varphi_{2\text{exp\textbackslash{} peak},t}$	0.0025***	0.0065***	−0.0010	−0.0008***	−0.0026*	0.0007*	0.0001
	(0.0009)	(0.0018)	(0.0012)	(0.0002)	(0.0015)	(0.0004)	(0.0001)
$\varphi_{3\text{trough\textbackslash{} rec},\;t}$	0.0077	−0.0173**	0.0309***	−0.0005	−0.0092	0.0004	−0.0001
	(0.0054)	(0.0085)	(0.0099)	(0.0004)	(0.0075)	(0.0016)	(0.0004)
$\varphi_{4rir,t}$	0.0010	−0.0017	0.0023***	0.0000	0.0002	0.0001	−0.0001
	(0.0007)	(0.0015)	(0.0009)	(0.0001)	(0.0014)	(0.0003)	(0.0001)
$\varphi_{5hv,t}$	−0.0088***	−0.0072***	−0.0002	−0.0004*	−0.0031	0.0005	0.0004***
	(0.0015)	(0.0023)	(0.0015)	(0.0002)	(0.0019)	(0.0004)	(0.0002)
R^2^	0.9180	0.9036	0.9131	0.9589	0.8046	0.9158	0.9065
S.E. of regression	(0.0034)	(0.0381)	(0.0246)	(0.0034)	(0.0327)	(0.0077)	(0.0024)

Note: This table reports coefficients obtained from regressing the time series of total and each component of systematic risk ($u_{SYS,SOURCE,t}$) on a constant and indicators for each stage of the business cycle, periods of rising interest rates and high volatility: $u_{SYS,SOURCE,t}=\varphi_{\text{0}}+\varphi_{\text{1}}u_{SYS,SOURCE,t-1}+\varphi_{\text{2}}I_{exppeak,t}+\varphi_{\text{3}}I_{throughrec,t}+\varphi_{\text{4}}I_{rir,t}+\varphi_{\text{5}}I_{hv,t}+e_{i,t}$. *Significant at the 90% confidence level; **significant at the 95% confidence level **; significant at the 99% confidence level; *** All regressions were run with Newey-West robust standard errors to ensure valid statistical inference.

**Table 5:** Tests of transmission of shocks from different stages of the business cycle, rising interest rates and high volatility to different sources of systematic risk of the ESG portfolio (Trough and recession to expansion and peak).

Dependent variable: $u_{SYS,SOURCE,t}$	$u_{SYS,ESG,t}$	$u_{SYS,WI,t}$	$u_{SYS,VX,t}$	$u_{SYS,TR,t}$	$u_{SYS,}h_{VX_{t},WI_{t}}$	$u_{SYS,}h_{WI_{t},TR_{t}}$	$u_{SYS,}h_{VX_{t},TR_{t}}$
**Coefficient**							
$\varphi_{3\text{trough\textbackslash{} rec},\;t}$	0.0052	−0.0237***	0.0320***	0.0003	−0.0066	−0.0003	−0.0002
	(0.0054)	(0.0087)	(0.0100)	(0.0004)	(0.0077)	(0.0016)	(0.0004)
$\varphi_{3\text{rec\textbackslash{} peak},t}$	−0.0025***	−0.0065***	0.0010	0.0008***	0.0026*	−0.0007*	−0.0001
	(0.0009)	(0.0018)	(0.0012)	(0.0002)	(0.0015)	(0.0004)	(0.0001)
$\varphi_{4rir,t}$	0.0010	−0.0017	0.0023***	0.0000	0.0002	0.0001	−0.0001
	(0.0007)	(0.0015)	(0.0009)	(0.0001)	(0.0014)	(0.0003)	(0.0001)
$\varphi_{5hv,t}$	−0.0088***	−0.0072***	−0.0002	−0.0004*	−0.0031	0.0005	0.0004***
	(0.0015)	(0.0023)	(0.0015)	(0.0002)	(0.0019)	(0.0004)	(0.0002)
R^2^	0.9180	0.9036	0.9131	0.9589	0.8046	0.9158	0.9065
S.E. of regression	(0.0034)	(0.0381)	(0.0246)	(0.0034)	(0.0327)	(0.0077)	(0.0024)

Note: This table reports coefficients obtained from regressing the time series of total and each component of systematic risk ($u_{SYS,SOURCE,t}$) on a constant and indicators for each stage of the business cycle, periods of rising interest rates and high volatility: $u_{SYS,SOURCE,t}=\varphi_{\text{0}}+\varphi_{\text{1}}u_{SYS,SOURCE,t-1}+\varphi_{\text{2}}I_{throughrec,t}+\varphi_{\text{3}}I_{recpeak_{\text{2}},t}+\varphi_{\text{4}}I_{rir,t}+\varphi_{\text{5}}I_{hv,t}+e_{i,t}$. *Significant at the 90% confidence level; **significant at the 95% confidence level **; significant at the 99% confidence level; ***. All regressions were run with Newey-West robust standard errors to ensure valid statistical inference.

When we allow for transmission shocks from the different components of systematic risk represented by the variances and covariances among the three common factors, we find some interesting results. In the case of the World Index ($u_{\text{SYS},\text{WI},t}$) as a component of systematic risk, from Table 4 we observe the stage of expansion to peak ($\varphi_{2\text{exp\textbackslash{} peak},\;t}$) and trough recession ($\varphi_{3\text{trough\textbackslash{} rec},\;t}$) are statistically significant and the first coefficient has a positive sign and the second a negative sign which implies that in the expansive phase of the business cycle the World Index causes total systematic risk to increase but in the trough and recession phase the opposite happens. From Table 5, we can observe that for the World Index, the stage of recession to peak ($\varphi_{3\text{rec\textbackslash{} peak},\;t}$) is statistically significant and the coefficient has a negative sign which means that for this specific stage of the business cycle, the effect of the World Index as source of systematic risk to the ESG portfolio decreases. Finally, when volatility increases ($\varphi_{5hv,t}$), we observe a similar effect in which the effect of the World Index as sources of systematic risk of the ESG portfolio decreases.

The most interesting results are the ones obtained with the factor that acts as our proxy for investor sentiment (fear), the VIX index ($u_{\text{SYS},\text{VX},t}$), in the case of both the expansion stages of the business cycle expansion to peak (Table 4) recession to peak (Table 5) the coefficients are not statistically significant. However, when we look at the effect of the VIX in the stage of trough and recession ($\varphi_{3\text{trough\textbackslash{} rec},\;t}$) and rising interest rates ($\varphi_{4\text{rir},\;t}$), the coefficients for the transmission shocks are positive and highly significant. This means that in periods of high-risk aversion (namely rising interest rates, and the business cycle is in the stage of trough and recession) changes in investor sentiment (fear) become a significant source of systematic risk to the ESG portfolio. These results hold even when we control for changes in volatility ($\varphi_{5hv,t}$) where we can observe that the coefficient is not significant. This means that the VIX component is more sensitive to state dependency than to general volatility risk.

From Table 4, we can observe that when we analyze the Bloomberg Treasury Index ($u_{\text{SYS,TR},\;t}$) as a component of systematic risk that proxies the level of interest rates. This component is statistically significant in the expansion to peak (see Table 4) stage of the business cycle (and the coefficient exhibits a negative sign. This can be interpreted that from expansion to peak, the interest rate causes a decrease in the effect that the component of systematic risk derived from changes in interest rates has on the ESG portfolio. The opposite happens (see Table 5) from the recession to expansion stage of the business cycle in which the level of interest rates causes an increase in the systematic risk derived from changes in interest rates on the ESG portfolio. Additionally, the high volatility period ($\varphi_{5hv,t}$) is significant and with a negative sign in the coefficient, which can be interpreted that increasing volatility in tandem with changes in business cycles have an effect in the systematic risk derived from changes in interest rates.

Finally, from the results obtained in Tables 4 and 5, that the covariances among the different components of systematic risk (namely the World Index and the VIX index ($u_{SYS,}h_{VX_{t},WI_{t}}$), World Index and the Bloomberg Treasury Index ($u_{SYS,}h_{WI_{t},TR_{t}}$), are statistically significant in the expansion to peak (Table 4) and recession to peak (Table 5). In the first case, the covariance between the World Index and the VIX ($u_{SYS,}h_{VX_{t},WI_{t}}$), the interaction decreases (increases) systematic risk in the expansion to peak (recession to peak) phases. In the second case, the covariance between the World Index and the Bloomberg Treasury Index ($u_{SYS,}h_{WI_{t},TR_{t}}$), the interaction increases (decreases) systematic risk in the expansion to peak (recession to peak) phases. Finally, in the case of the covariance between the VIX and the Bloomberg Treasury Index and the VIX ($u_{SYS,}h_{VX_{t},TR_{t}}$) changes in volatility ($\varphi_{5hv,t}$) increase systematic risk. We do this to control transmission shocks derived from the interaction of the common factors to changes in the business cycle. We conclude that the effect of interaction among the different factors as a source of systematic risk to the ESG portfolio is significant only in the expansion phases of the business cycle.

## Robustness test

To test the robustness of our results, we ran the proposed model replacing the S&P 500 ESG index with the MSCI World Top ESG Select Index which exhibits lower correlation with the World Index and has higher exclusionary ESG standards than the S&P 500 ESG index, for the methodology see: [39]. The results obtained in Table 6 and 7 show that total systematic risk ($u_{\text{SYS},\text{ESG},t}$) does affect the ESG portfolio when exposed to changes in the business cycle, rising interest rates or change in volatility. However, when we test different components of systematic risk, we find some interesting results. First, in the case of the World Index ($u_{\text{SYS},\text{WI},t}$) as a source of systematic risk it shows a positive correlation with periods of high interest rates and high volatility. Interestingly, the relationship between the business cycles and investor sentiment (fear) as a source of systematic risk proxied by the VIX ($u_{\text{SYS},\text{VX},t}$) is significant in the expansion to peak, trough and recession, and recession to peak. In the recession to peak ($\varphi_{3\text{rec\textbackslash{} peak},\;t}$), the negative coefficient is signaling that investor sentiment (fear) decreases in the expansive phase of the economy.

**Table 6:** Robustness tests of transmission of shocks from different stages of the business cycle, rising interest rates and high volatility to different sources of systematic risk of the ESG portfolio (expansion to peak to trough and recession).

Dependent variable: $u_{SYS,SOURCE,t}$	$u_{SYS,ESG,t}$	$u_{SYS,WI,t}$	$u_{SYS,VX,t}$	$u_{SYS,TR,t}$	$u_{SYS,}h_{VX_{t},WI_{t}}$	$u_{SYS,}h_{WI_{t},TR_{t}}$	$u_{SYS,}h_{VX_{t},TR_{t}}$
**Coefficient**							
$\varphi_{2\text{exp\textbackslash{} peak},t}$	0.0005	0.0009	0.0021***	−0.0009***	−0.0011*	−0.0003	−0.0003
	(0.0011)	(0.0010)	(0.0006)	(0.0004)	(0.0007)	(0.0002)	(0.0002)
$\varphi_{3\text{trough\textbackslash{} rec},\;t}$	−0.0003	−0.0023	0.0181*	−0.0014	−0.0061	−0.0031	−0.0031
	(0.0090)	(0.0043)	(0.0097)	(0.0020)	(0.0047)	(0.0023)	(0.0023)
$\varphi_{4rir,t}$	0.0012	0.0016*	0.0006	−0.0002	−0.0003	−0.0003	−0.0003
	(0.0010)	(0.0009)	(0.0007)	(0.0002)	(0.0006)	(0.0002)	(0.0002)
$\varphi_{5hv,t}$	0.0019	0.0023*	0.0001	−0.0001	−0.0007	0.0005*	0.0005*
	(0.0014)	(0.0013)	(0.0006)	(0.0004)	(0.0008)	(0.0002)	(0.0002)
R^2^	0.9504	0.9560	0.8586	0.9439	0.6088	0.6304	0.6304
S.E. of regression	(0.0072)	(0.0218)	(0.0141)	(0.0072)	(0.0132)	(0.0044)	(0.0044)

Note: This table reports coefficients obtained from regressing the time series of total and each component of systematic risk ($u_{SYS,SOURCE,t}$) on a constant and indicators for each stage of the business cycle, periods of rising interest rates and high volatility: $u_{SYS,SOURCE,t}=\varphi_{\text{0}}+\varphi_{\text{1}}u_{SYS,SOURCE,t-1}+\varphi_{\text{2}}I_{exppeak,t}+\varphi_{\text{3}}I_{throughrec,t}+\varphi_{\text{4}}I_{rir,t}+\varphi_{\text{5}}I_{hv,t}+e_{i,t}$. *Significant at the 90% confidence level; **significant at the 95% confidence level **; significant at the 99% confidence level; ***. All regressions were run with Newey-West robust standard errors to ensure valid statistical inference.

**Table 7:** Robustness tests of transmission of shocks from different stages of the business cycle, rising interest rates and high volatility to different sources of systematic risk of the ESG portfolio (Trough and recession to expansion and peak).

Dependent variable: $u_{SYS,SOURCE,t}$	$u_{SYS,ESG,t}$	$u_{SYS,WI,t}$	$u_{SYS,VX,t}$	$u_{SYS,TR,t}$	$u_{SYS,}h_{VX_{t},WI_{t}}$	$u_{SYS,}h_{WI_{t},TR_{t}}$	$u_{SYS,}h_{VX_{t},TR_{t}}$
**Coefficient**							
$\varphi_{\text{2trough\textbackslash{} rec},\;t}$	−0.0008	−0.0031	0.0160*	−0.0005	−0.0017	−0.0017	−0.0028
	(0.0091)	(0.0044)	(0.0096)	(0.0020)	(0.0021)	(0.0021)	(0.0022)
$\varphi_{3\text{rec\textbackslash{} peak},t}$	−0.0005	−0.0009	−0.0021***	0.0009***	−0.0007*	−0.0007*	0.0003
	(0.0011)	(0.0010)	(0.0006)	(0.0004)	(0.0004)	(0.0004)	(0.0002)
$\varphi_{4rir,t}$	0.0012	0.0016*	0.0006	−0.0002	−0.0002	−0.0002	−0.0003
	(0.0010)	(0.0009)	(0.0007)	(0.0002)	(0.0002)	(0.0002)	(0.0002)
$\varphi_{5hv,t}$	0.0019	0.0023*	0.0001	−0.0001	−0.0001	−0.0001	0.0005*
	(0.0014)	(0.0013)	(0.0006)	(0.0004)	(0.0004)	(0.0004)	(0.0002)
R^2^	0.9504	0.9560	0.8586	0.9439	0.9003	0.9003	0.6304
S.E. of regression	(0.0072)	(0.0218)	(0.0141)	(0.0072)	(0.0076)	(0.0076)	(0.0044)

Note: This table reports coefficients obtained from regressing the time series of total and each component of systematic risk ($u_{SYS,SOURCE,t}$) on a constant and indicators for each stage of the business cycle, periods of rising interest rates and high volatility: $u_{SYS,SOURCE,t}=\varphi_{\text{0}}+\varphi_{\text{1}}u_{SYS,SOURCE,t-1}+\varphi_{\text{2}}I_{throughrec,t}+\varphi_{\text{3}}I_{recpeak_{\text{2}},t}+\varphi_{\text{4}}I_{rir,t}+\varphi_{\text{5}}I_{hv,t}+e_{i,t}$. *Significant at the 90% confidence level; **significant at the 95% confidence level **; significant at the 99% confidence level; ***. All regressions were run with Newey-West robust standard errors to ensure valid statistical inference.

Finally, from the results obtained in Tables 6 and 7, the covariances among the different components of systematic risk (namely the World Index and the VIX index ($u_{SYS,}h_{VX_{t},WI_{t}}$) is statistically significant in the expansion to peak and in the recession to peak and in both cases the interaction between the factors decreases systematic risk. In the case of the World Index and the Bloomberg Treasury Index ($u_{SYS,}h_{WI_{t},TR_{t}}$), the relationship is statistically significant in the recession to peak (Table 7) and in high volatility periods (Table 6). Finally, the covariance between VIX and the Bloomberg Treasury Index ($u_{SYS,}h_{VX_{t},TR_{t}}$), the only driver for this component of systematic risk is the high volatility period ($\varphi_{5hv,t}$) which causes an increase in systematic risk.

## Conclusions

In the present study, we find evidence of the time varying nature of investment sentiment (fear) through different stages of the business cycle and in periods of tightening monetary policy. In particular, the effects of investment sentiment (fear) proxied by the VIX index and its effect on the systematic risk of an ESG portfolio proxied by the S&P ESG index. One important result is that in the expansion phases of the business cycle, investment sentiment (fear) as a source of systematic risk, is not a statistically significant source of systematic risk. However, the opposite is true in recession and trough period in times of rising interest rates. Finally, in the stage of recession to peak, which are usually stages where the interest rate decreases, the systematic risk decreases and the unique risk of the ESG portfolio increases, being both statistically significant. When we run the appropriate robustness test to further isolate the ESG effect, the role of investor sentiment (fear) in the through and recession hold even when controlling for high volatility. The main conclusion is that the effect of investor sentiment (fear) proxied by the systematic risk of an ESG portfolio is irrelevant on the expansion phases of the economy but in the contracting phases becomes highly relevant which in turn can explain the conflicting evidence around the portfolio performance of ESG investments due to overinvesting in times of low interest rates and overselling in times of monetary tightening.

## Supporting information

S1 File Asgnewdata. (XLSX)
