# Supplementary material for: The effects on investor sentiment and business cycles on the systematic risk of an ESG portfolio
Source: PLoS One. 2026 Nov 5;21(11):e0341348. doi: 10.1371/journal.pone.0341348 (PMC13739132; doi:10.1371/journal.pone.0341348)
Supplement: Peer review history

# Peer review: Decision Letter 0

**DOI:** 10.1371/journal.pone.0341348.r001

**Authors:** Iván Barreda-Tarrazona

28 May 2026

Dear Dr. Cayon-Fallon,

Please submit your revised manuscript by Jul 12 2026 11:59PM. If you will need more time than this to complete your revisions, please reply to this message or contact the journal office at plosone@plos.org. When you're ready to submit your revision, log on to https://www.editorialmanager.com/pone/ and select the 'Submissions Needing Revision' folder to locate your manuscript file.

- A letter that responds to each point raised by the academic editor and reviewer(s). You should upload this letter as a separate file labeled 'Response to Reviewers'.
- A marked-up copy of your manuscript that highlights changes made to the original version. You should upload this as a separate file labeled 'Revised Manuscript with Track Changes'.
- An unmarked version of your revised paper without tracked changes. You should upload this as a separate file labeled 'Manuscript'.

If you would like to make changes to your financial disclosure, please include your updated statement in your cover letter. Guidelines for resubmitting your figure files are available below the reviewer comments at the end of this letter.

If applicable, we recommend that you deposit your laboratory protocols in protocols.io to enhance the reproducibility of your results. Protocols.io assigns your protocol its own identifier (DOI) so that it can be cited independently in the future. For instructions see: https://journals.plos.org/plosone/s/submission-guidelines#loc-laboratory-protocols. Additionally, PLOS ONE offers an option for publishing peer-reviewed Lab Protocol articles, which describe protocols hosted on protocols.io. Read more information on sharing protocols at https://plos.org/protocols?utm_medium=editorial-email&utm_source=authorletters&utm_campaign=protocols.

As the corresponding author, your ORCID iD is verified in the submission system and will appear in the published article. PLOS supports the use of ORCID, and we encourage all coauthors to register for an ORCID iD and use it as well. Please encourage your coauthors to verify their ORCID iD within the submission system before final acceptance, as unverified ORCID iDs will not appear in the published article. *Only*  the individual author can complete the verification step; PLOS staff *cannot*  verify ORCID iDs on behalf of authors.

We look forward to receiving your revised manuscript.

Kind regards,

Iván Barreda-Tarrazona, PhD

Academic Editor

PLOS One

**Journal Requirements:**

When submitting your revision, we need you to address these additional requirements.

1. Please ensure that your manuscript meets PLOS ONE's style requirements, including those for file naming. The PLOS ONE style templates can be found at

https://journals.plos.org/plosone/s/file?id=wjVg/PLOSOne_formatting_sample_main_body.pdf and

https://journals.plos.org/plosone/s/file?id=ba62/PLOSOne_formatting_sample_title_authors_affiliations.pdf

2. Please remove your figures from within your manuscript file, leaving only the individual TIFF/EPS image files, uploaded separately. These will be automatically included in the reviewers’ PDF.

3. If tables are embedded in the manuscript and ALSO loaded as separate files, please delete the separate files.

4. If the reviewer comments include a recommendation to cite specific previously published works, please review and evaluate these publications to determine whether they are relevant and should be cited. There is no requirement to cite these works unless the editor has indicated otherwise.

[Note: HTML markup is below. Please do not edit.]

Reviewers' comments:

Reviewer's Responses to Questions

**Comments to the Author**

1. Is the manuscript technically sound, and do the data support the conclusions?

Reviewer #1: Yes

Reviewer #2: Partly

**********

2. Has the statistical analysis been performed appropriately and rigorously?

Reviewer #1: Yes

Reviewer #2: Yes

**********

3. Have the authors made all data underlying the findings in their manuscript fully available?

The PLOS Data policy

Reviewer #1: Yes

Reviewer #2: Yes

**********

4. Is the manuscript presented in an intelligible fashion and written in standard English?

Reviewer #1: Yes

Reviewer #2: Yes

**********

Reviewer #1: This paper studies the time-varying systematic risk of an ESG portfolio and relates it to macroeconomic conditions, with particular emphasis on business cycle phases and periods of monetary tightening. The empirical analysis is based on daily data over 2010–2024 and models ESG returns jointly with global equity markets, implied volatility, and interest rates in a multivariate GARCH framework. The authors then decompose ESG volatility into systematic and idiosyncratic components and examine how their relative importance varies across economic states.

The main result is that the contribution of the volatility-related factor (proxied by the VIX) to ESG systematic risk is not constant. It appears limited during expansion periods, but becomes more pronounced during downturns and episodes of rising interest rates, while exposure to global market risk remains persistent across regimes.

The question the paper addresses is a natural one. ESG investing is often discussed in terms that implicitly assume stable risk properties, yet both theory and evidence suggest that the relevance of different risk components tends to vary with economic conditions. In that sense, the paper’s contribution lies in documenting that ESG portfolios are not immune to this broader pattern: their exposure to aggregate risk factors, particularly those linked to uncertainty, appears to shift in ways that are aligned with the macroeconomic environment.

At the same time, the empirical design is layered, and the interpretation of the results depends critically on how these layers interact. Some of the paper’s conclusions are best understood as describing conditional variation in estimated risk components, rather than as identifying a distinct underlying mechanism. Clarifying this distinction would strengthen the contribution.

Here are some main point that in my view need to be adressed:

1. Interpretation of the VIX

A central element of the paper is the interpretation of the VIX as a proxy for investor sentiment. This interpretation is intuitive, but it is not fully pinned down by the empirical design.

The VIX is a measure of expected market volatility derived from option prices and reflects a combination of uncertainty, demand for protection, and variation in risk premia. The finding that VIX-related exposure becomes more important in recessions and tightening periods is therefore consistent with a broader pattern: in adverse states, uncertainty and downside risk become more salient, and assets tend to respond more strongly to these conditions.

The empirical results clearly show that the VIX component is state-dependent, but they do not isolate whether this reflects sentiment in a narrow sense or more general volatility risk. A more precise framing—emphasizing aggregate uncertainty or risk conditions—would better align the interpretation with the evidence, or alternatively using additional proxies.

2.Second-stage regressions and interpretation of coefficients

The core results are obtained by regressing estimated systematic-risk components (derived from the GARCH model) on regime dummies and lagged dependent variables. This structure raises two related issues:first, the dependent variables in these regressions are generated quantities from a first-stage estimation. The paper treats them as observed variables, but does not adjust inference for estimation error in the first stage. This is common in practice, but it implies that the reported standard errors may understate uncertainty.

Second, the inclusion of a lagged dependent variable captures persistence, but also means that much of the variation in the dependent variable is mechanically explained. The relatively high R^2 values should therefore not be interpreted as strong explanatory power of the regime variables themselves.

These points do not invalidate the results, but they do affect how they should be interpreted. The regression evidence is best understood as showing that estimated risk shares co-move with macroeconomic regimes, rather than providing a precise decomposition of causal effects.

3. Statistical inference and time-series properties

Relatedly, the paper does not clearly specify whether time-series robust standard errors are used in the second-stage regressions. Given the use of daily data and highly persistent volatility-related measures, this is important.

Without appropriate corrections (e.g., HAC or Newey-West), standard errors may be biased downward, which could affect statistical significance. This is particularly relevant for results that are interpreted as regime-specific differences.

4. Definition of economic regimes

The paper relies on NBER business cycle classifications to define economic states. While this is a natural approach, in the sample considered the recession period is very short and largely driven by the COVID episode.

This has two implications. First, the statistical pwer of the recession regime is limited. Second, the results for this regime may be influenced by a single, highly unusual event. Given that the paper’s main contribution relies on differences across regimes, this is a non-trivial concern.

More generally, periods in which uncertainty becomes particularly salient tend to be concentrated and episodic. This makes it difficult to distinguish between a general pattern and an event-specific effect. It would therefore be useful to examine robustness to alternative definitions of economic conditions, for example using continuous measures or volatility-based regimes.

5. ESG versus standard risk exposures

The analysis does not fully account for portfolio composition and standard factor exposures. ESG indices often exhibit tilts toward particular sectors or firm characteristics, which can influence sensitivity to macroeconomic variables.

In particular, environments in which discount rates change rapidly tend to affect certain types of firms more strongly. In such cases, what appears as a change in ESG risk exposure may reflect broader valuation adjustments rather than ESG-specific mechanisms.

The current specification does not disentangle these effects. Comparing ESG returns to the parent index or incorporating standard factor controls would help clarify whether the results are specific to ESG screening.

6. Positioning of the contribution

The contribution of the paper would be clearer if it were framed more narrowly.

The strongest result is that the coposition of ESG systematic risk is state-dependent, and in particular that sensitivity to volatility-related factors increases in periods when uncertainty and downside risk are more prominent. Emphasizing this mechanism would sharpen the overall narrative and align the claims more closely with the empirical evidence.

Minor issues

The paper is generally clearly written, but a number of smaller points could be addressed:

• The terms “sentiment”, “fear”, “risk aversion”, and “volatility” are sometimes used interchangeably, although they refer to distinct concepts.

• The interpretation of the Treasury index as a proxy for interest rates could be clarified.

• Business cycle terminology and notation could be made more consistent.

• The econometric specification is difficult to follow in places due to notation and formatting.

• The discussion focuses primarily on statistical significance; more attention to economic magnitudes would be helpful.

• Figures could be made more informative, particularly regarding the decomposition of risk components.

• There are a number of minor typos

Reviewer #2: This manuscript explores the relationship between investor sentiment, business cycles, and the systematic risk of ESG portfolios. The topic is timely and relevant given the rapid growth of ESG investing and recent monetary tightening cycles, making the study a meaningful contribution to the fields of asset pricing and sustainable finance. The manuscript employs an M-GARCH framework to analyze the dynamic effects of various risk sources on ESG portfolios, thereby adding methodological depth. While the study shows potential and provides a relatively comprehensive literature review, the authors are encouraged to strengthen the economic interpretation of the results further, provide stronger justification for the model specification, and conduct additional robustness checks to enhance the credibility and persuasiveness of the analysis. I recommend a minor revision to improve the manuscript's overall quality and suitability for publication. Suggestions for improvement are provided below:

1.

The residuals obtained from AR models are essentially the statistically unexplained components of the series. However, this interpretation does not fully correspond to the economic meaning of idiosyncratic risk in asset pricing theory. In general, idiosyncratic risk refers to asset-specific risk that is unrelated to common market factors or systematic influences. Therefore, the manuscript would benefit from providing stronger theoretical justification for treating AR residuals as proxies for idiosyncratic risk within the proposed framework.

2.

The manuscript states that risk aversion, proxied by the VIX index, has a significant effect on the systematic risk of ESG portfolios during economic downturns. However, this conclusion appears to be more consistent with statistical association rather than strict causal inference. While the empirical results suggest a significant dynamic relationship between the variables, the current modeling framework may not be sufficient to establish a direct causal effect. The authors are encouraged to clarify the interpretation of the results.

3.

The authors are encouraged to provide additional robustness analyses across different scenarios, such as using non-ESG portfolios as benchmarks or employing alternative ESG indices and portfolio constructions. Such analyses would help confirm that the findings are not solely dependent on a specific dataset, sample period, or model specification.

**********

what does this mean?). If published, this will include your full peer review and any attached files.

If you choose “no”, your identity will remain anonymous but your review may still be made public.

**Do you want your identity to be public for this peer review?**  For information about this choice, including consent withdrawal, please see our Privacy Policy

Reviewer #1: No

Reviewer #2: No

**********

[NOTE: If reviewer comments were submitted as an attachment file, they will be attached to this email and accessible via the submission site. Please log into your account, locate the manuscript record, and check for the action link "View Attachments". If this link does not appear, there are no attachment files.]

To ensure your figures meet our technical requirements, please review our figure guidelines: https://journals.plos.org/plosone/s/figures

You may also use PLOS’s free figure tool, NAAS, to help you prepare publication quality figures: https://journals.plos.org/plosone/s/figures#loc-tools-for-figure-preparation.

NAAS will assess whether your figures meet our technical requirements by comparing each figure against our figure specifications.

# Author comment: Author response to Decision Letter 1

**DOI:** 10.1371/journal.pone.0341348.r002

6 Jul 2026

We thank both reviewers for their valuable comments, which helped strengthen the validity and robustness of our results. Our responses to their comments and suggestions are provided below:

Answers to reviewer number 1:

1. Interpretation of the VIX

A central element of the paper is the interpretation of the VIX as a proxy for investor sentiment. This interpretation is intuitive, but it is not fully pinned down by the empirical design.

The VIX is a measure of expected market volatility derived from option prices and reflects a combination of uncertainty, demand for protection, and variation in risk premia. The finding that VIX-related exposure becomes more important in recessions and tightening periods is therefore consistent with a broader pattern: in adverse states, uncertainty and downside risk become more salient, and assets tend to respond more strongly to these conditions.

The empirical results clearly show that the VIX component is state-dependent, but they do not isolate whether this reflects sentiment in a narrow sense or more general volatility risk. A more precise framing—emphasizing aggregate uncertainty or risk conditions—would better align the interpretation with the evidence, or alternatively using additional proxies.

We thank the reviewer for the comment and in this version, we added an additional proxy for high volatility regimes using the VIX value, the new proxy is incorporated in Tables 4 and 5, and the corrected paragraph is as follows:

Also, for robustness purposes and to examine the effects of changes in volatility regimes, we constructed a dummy variable that takes the value of 1 when the VIX is above the accepted threshold for high volatility and 0 otherwise. We used the threshold value of 23.81, following Chen & Yang (2021), who found that this is the cut-off point at which most active VIX futures traders, both short and long, change their investment strategies. Therefore, when the VIX index is above 23.81, it is considered a high-volatility regime; when it is below this threshold, it is considered a low-volatility regime.

2.Second-stage regressions and interpretation of coefficients

The core results are obtained by regressing estimated systematic-risk components (derived from the GARCH model) on regime dummies and lagged dependent variables. This structure raises two related issues: first, the dependent variables in these regressions are generated quantities from a first-stage estimation. The paper treats them as observed variables, but does not adjust inference for estimation error in the first stage. This is common in practice, but it implies that the reported standard errors may understate uncertainty.

Second, the inclusion of a lagged dependent variable captures persistence, but also means that much of the variation in the dependent variable is mechanically explained. The relatively high R^2 values should therefore not be interpreted as strong explanatory power of the regime variables themselves.

These points do not invalidate the results, but they do affect how they should be interpreted. The regression evidence is best understood as showing that estimated risk shares co-move with macroeconomic regimes, rather than providing a precise decomposition of causal effects.

The regressions were run using Newey-West robust standard errors to ensure valid statistical inference. However, in the first version, we forgot to include the corresponding annotations. We ran the new regressions using the new proxy for aggregate volatility (see Tables 4, 5, 6, and 7), as well as an additional robustness test to further isolate the ESG effect and validate the macroeconomic regimes. The paragraphs describing the robustness test are as follows:

To test the robustness of our results, we ran the proposed model replacing the S&P 500 ESG index with the MSCI World Top ESG Select Index which exhibits lower correlation with the World Index and has higher exclusionary ESG standards than the S&P 500 ESG index . The results obtained in Table 6 and 7 show that total systematic risk ( ) does affect the ESG portfolio when exposed to changes in the business cycle, rising interest rates or change in volatility. However, when we test different components of systematic risk, we find some interesting results. First, in the case of the World Index ( ) as a source of systematic risk it shows a positive correlation with periods of high interest rates and high volatility. Interestingly, the relationship between the business cycles and investor sentiment (fear) as a source of systematic risk proxied by the VIX ( ) is significant in the expansion to peak, trough and recession, and recession to peak. In the recession to peak ( ), the negative coefficient is signaling that investor sentiment (fear) decreases in the expansive phase of the economy.

Finally, from the results obtained in tables 6 and 7, the covariances among the different components of systematic risk (namely the World Index and the VIX index ( ) is statistically significant in the expansion to peak and in the recession to peak and in both cases the interaction between the factors decreases systematic risk. In the case of the World Index and the Bloomberg Treasury Index ( ), the relationship is statistically significant in the recession to peak (Table 7) and in high volatility periods (Table 6). Finally, the covariance between VIX and the Bloomberg Treasury Index ( ), the only driver for this component of systematic risk is the high volatility period ( ) which causes an increase in systematic risk.

3. Statistical inference and time-series properties

Relatedly, the paper does not clearly specify whether time-series robust standard errors are used in the second-stage regressions. Given the use of daily data and highly persistent volatility-related measures, this is important.

Without appropriate corrections (e.g., HAC or Newey-West), standard errors may be biased downward, which could affect statistical significance. This is particularly relevant for results that are interpreted as regime-specific differences.

The regressions were run using Newey-West robust standard errors to ensure valid statistical inference. However, in the first version, we forgot to include the appropriate annotations. We ran the new regressions using the new proxy for aggregate volatility (see Tables 4, 5, 6, and 7), and the table notes now mention the Newey-West correction.

4. Definition of economic regimes

The paper relies on NBER business cycle classifications to define economic states. While this is a natural approach, in the sample considered the recession period is very short and largely driven by the COVID episode.

This has two implications. First, the statistical power of the recession regime is limited. Second, the results for this regime may be influenced by a single, highly unusual event. Given that the paper’s main contribution relies on differences across regimes, this is a non-trivial concern.

More generally, periods in which uncertainty becomes particularly salient tend to be concentrated and episodic. This makes it difficult to distinguish between a general pattern and an event-specific effect. It would therefore be useful to examine robustness to alternative definitions of economic conditions, for example using continuous measures or volatility-based regimes.

We thank the reviewer for this comment. In this version, we added an additional proxy for high-volatility regimes using the VIX value, since changes in the volatility regime are not state-dependent under this new proxy. As the reviewer mentioned, this also helps disentangle the effects attributable to regimes and volatility. The new proxy is incorporated in Tables 4, 5, 6, and 7, as well as in the revised interpretation of the results. The most relevant changes are as follows:

Finally, when volatility increases ( ), we observe a similar effect in which the effect of the World Index as sources of systematic risk of the ESG portfolio decreases……

….These results hold even when we control for changes in volatility ( ) where we can observe that the coefficient is not significant. This means that the VIX component is more sensitive…

…Additionally, the high volatility period ( ) is significant and with a negative sign in the coefficient, which can be interpreted that increasing volatility in tandem with changes in business cycles have an effect in the systematic risk derived from changes in interest rates.

5. ESG versus standard risk exposures

The analysis does not fully account for portfolio composition and standard factor exposures. ESG indices often exhibit tilts toward particular sectors or firm characteristics, which can influence sensitivity to macroeconomic variables.

In particular, environments in which discount rates change rapidly tend to affect certain types of firms more strongly. In such cases, what appears as a change in ESG risk exposure may reflect broader valuation adjustments rather than ESG-specific mechanisms.

The current specification does not disentangle these effects. Comparing ESG returns to the parent index or incorporating standard factor controls would help clarify whether the results are specific to ESG screening.

This is a valuable comment. In order to disentangle the ESG effect, we conducted a robustness test, which reads as follows:

To test the robustness of our results, we ran the proposed model replacing the S&P 500 ESG index with the MSCI World Top ESG Select Index which exhibits lower correlation with the World Index and has higher exclusionary ESG standards than the S&P 500 ESG index . The results obtained in Table 6 and 7 show that total systematic risk ( ) does affect the ESG portfolio when exposed to changes in the business cycle, rising interest rates or change in volatility. However, when we test different components of systematic risk, we find some interesting results. First, in the case of the World Index ( ) as a source of systematic risk it shows a positive correlation with periods of high interest rates and high volatility. Interestingly, the relationship between the business cycles and investor sentiment (fear) as a source of systematic risk proxied by the VIX ( ) is significant in the expansion to peak, trough and recession, and recession to peak. In the recession to peak ( ), the negative coefficient is signaling that investor sentiment (fear) decreases in the expansive phase of the economy.

Finally, from the results obtained in tables 6 and 7, the covariances among the different components of systematic risk (namely the World Index and the VIX index ( ) is statistically significant in the expansion to peak and in the recession to peak and in both cases the interaction between the factors decreases systematic risk. In the case of the World Index and the Bloomberg Treasury Index ( ), the relationship is statistically significant in the recession to peak (Table 7) and in high volatility periods (Table 6). Finally, the covariance between VIX and the Bloomberg Treasury Index ( ), the only driver for this component of systematic risk is the high volatility period ( ) which causes an increase in systematic risk.

6. Positioning of the contribution

The contribution of the paper would be clearer if it were framed more narrowly.

The strongest result is that the coposition of ESG systematic risk is state-dependent, and in particular that sensitivity to volatility-related factors increases in periods when uncertainty and downside risk are more prominent. Emphasizing this mechanism would sharpen the overall narrative and align the claims more closely with the empirical evidence.

We thank the reviewer for this suggestion. In the new version, we have reinforced this specific point in both the interpretation of the results and the conclusion.

Minor issues

The paper is generally clearly written, but a number of smaller points could be addressed:

• The terms “sentiment”, “fear”, “risk aversion”, and “volatility” are sometimes used interchangeably, although they refer to distinct concepts.

When possible, we tried to refer to investor sentiment (fear) throughout this new version.

• The interpretation of the Treasury index as a proxy for interest rates could be clarified.

We did it and the clarification reads:

This is important because this index is designed to capture changes in market interest rates and reduce jumps in short-term volatility, in other words the index reflects the immediate impact of interest rates in the market.

• Business cycle terminology and notation could be made more consistent.

We revised the notation for coherence.

• The econometric specification is difficult to follow in places due to notation and formatting.

We revised the notation for coherence.

• The discussion focuses primarily on statistical significance; more attention to economic magnitudes would be helpful.

We tried to center more discussion around the changes in economic regimes

• Figures could be made more informative, particularly regarding the decomposition of risk components.

We opted for just using total systematic risk to make the figure clearer.

• There are a number of minor typos

We corrected some typos.

Answers to reviewer number 2:

Reviewer #2: This manuscript explores the relationship between investor sentiment, business cycles, and the systematic risk of ESG portfolios. The topic is timely and relevant given the rapid growth of ESG investing and recent monetary tightening cycles, making the study a meaningful contribution to the fields of asset pricing and sustainable finance. The manuscript employs an M-GARCH framework to analyze the dynamic effects of various risk sources on ESG portfolios, thereby adding methodological depth. While the study shows potential and provides a relatively comprehensive literature review, the authors are encouraged to strengthen the economic interpretation of the results further, provide stronger justification for the model specification, and conduct additional robustness checks to enhance the credibility and persuasiveness of the analysis. I recommend a minor revision to improve the manuscript's overall quality and suitability for publication. Suggestions for improvement are provided below:

1. The residuals obtained from AR models are essentially the statistically unexplained components of the series. However, this interpretation does not fully correspond to the economic meaning of idiosyncratic risk in asset pricing theory. In general, idiosyncratic risk refers to asset-specific risk that is unrelated to common market factors or systematic influences. Therefore, the manuscript would benefit from providing stronger theoretical justification for treating AR residuals as proxies for idiosyncratic risk within the proposed framework.

This is an important point, and we added a footnote referring readers to the seminal paper in which risk decomposition is explained. The footnote reads as follows:

For the theoretical explanation of how the beta is decomposed into systematic and idiosyncratic risk please refer to Sharpe (1964).

2. The manuscript states that risk aversion, proxied by the VIX index, has a significant effect on the systematic risk of ESG portfolios during economic downturns. However, this conclusion appears to be more consistent with statistical association rather than strict causal inference. While the empirical results suggest a significant dynamic relationship between the variables, the current modeling framework may not be sufficient to establish a direct causal effect. The authors are encouraged to clarify the interpretation of the results.

We have clarified the conclusions and added another proxy for volatility. All regressions were run using Newey-West robust standard errors to ensure valid statistical inference in both the initial empirical design and the robustness test. The revised conclusion reads as follows:

… However, the opposite is true in recession and trough period in times of rising interest rates. Finally, in the stage of recession to peak, which are usually stages where the interest rate decreases, the systematic risk decreases and the unique risk of the ESG portfolio increases, being both statistically significant. When we run the appropriate robustness test to further isolate the ESG effect, the role of investor sentiment (fear) in the through and recession hold even when controlling for high volatility. The main conclusion is that the effect of investor sentiment (fear) proxied by the systematic risk of an ESG portfolio is irrelevant on the expansion phases of the economy but in the contracting phases becomes highly relevant which in turn can explain the conflicting evidence around the portfolio performance of ESG investments due to overinvesting in times of low interest rates and overselling in times of monetary tightening…

3. The authors are encouraged to provide additional robustness analyses across different scenarios, such as using non-ESG portfolios as benchmarks or employing alternative ESG indices and portfolio constructions. Such analyses would help confirm that the findings are not solely dependent on a specific dataset, sample period, or model specification.

In this version, we added an additional proxy for high volatility and an alternative portfolio to further isolate the ESG effect. The revised version reads as follows:

To test the robustness of our results, we ran the proposed model replacing the S&P 500 ESG index with the MSCI World Top ESG Select Index which exhibits lower correlation with the World Index and has higher exclusionary ESG standards than the S&P 500 ESG index . The results obtained in Table 6 and 7 show that total systematic risk ( ) does affect the ESG portfolio when exposed to changes in the business cycle, rising interest rates or change in volatility. However, when we test different components of systematic risk, we find some interesting results. First, in the case of the World Index ( ) as a source of systematic risk it shows a positive correlation with periods of high interest rates and high volatility. Interestingly, the relationship between the business cycles and investor sentiment (fear) as a source of systematic risk proxied by the VIX ( ) is significant in the expansion to peak, trough and recession, and recession to peak. In the recession to peak ( ), the negative coefficient is signaling that investor sentiment (fear) decreases in the expansive phase of the economy.

Finally, from the results obtained in tables 6 and 7, the covariances among the different components of systematic risk (namely the World Index and the VIX index ( ) is statistically significant in the expansion to peak and in the recession to peak and in both cases the interaction between the factors decreases systematic risk. In the case of the World Index and the Bloomberg Treasury Index ( ), the relationship is statistically significant in the recession to peak (Table 7) and in high volatility periods (Table 6). Finally, the covariance between VIX and the Bloomberg Treasury Index ( ), the only driver for this component of systematic risk is the high volatility period ( ) which causes an increase in systematic risk.

Attachment Submitted filename: Response to reviewers.docx

# Peer review: Decision Letter 1

**DOI:** 10.1371/journal.pone.0341348.r003

**Authors:** Iván Barreda-Tarrazona

14 Oct 2026

The Effects on Investor Sentiment and Business Cycles on the Systematic Risk of an ESG Portfolio

PONE-D-26-00622R1

Dear Dr. Cayon-Fallon,

We’re pleased to inform you that your manuscript has been judged scientifically suitable for publication and will be formally accepted for publication once it meets all outstanding technical requirements.

Within one week, you’ll receive an e-mail detailing the required amendments. When these have been addressed, you’ll receive a formal acceptance letter and your manuscript will be scheduled for publication.

An invoice will be generated when your article is formally accepted. Please note, if your institution has a publishing partnership with PLOS and your article meets the relevant criteria, all or part of your publication costs will be covered. Please make sure your user information is up-to-date by logging into Editorial Manager at Editorial Manager® and clicking the ‘Update My Information' link at the top of the page. For questions related to billing, please contact billing support.

If your institution or institutions have a press office, please notify them about your upcoming paper to help maximize its impact. If they’ll be preparing press materials, please inform our press team as soon as possible -- no later than 48 hours after receiving the formal acceptance. Your manuscript will remain under strict press embargo until 2 pm Eastern Time on the date of publication. For more information, please contact onepress@plos.org.

Kind regards,

Iván Barreda-Tarrazona, PhD

Academic Editor

PLOS One

Additional Editor Comments (optional):

Reviewers' comments:

Reviewer's Responses to Questions

**Comments to the Author**

Reviewer #1: All comments have been addressed

Reviewer #2: All comments have been addressed

**********

2. Is the manuscript technically sound, and do the data support the conclusions?

Reviewer #1: Yes

Reviewer #2: Yes

**********

3. Has the statistical analysis been performed appropriately and rigorously?

Reviewer #1: Yes

Reviewer #2: Yes

**********

4. Have the authors made all data underlying the findings in their manuscript fully available?

The PLOS Data policy

Reviewer #1: Yes

Reviewer #2: Yes

**********

5. Is the manuscript presented in an intelligible fashion and written in standard English?

Reviewer #1: Yes

Reviewer #2: Yes

**********

Reviewer #1: One only comment at this stage: The additional robustness analysis using the MSCI World Top ESG Select Index is a useful improvement, as it helps establish whether the findings hold across different ESG portfolios. However, I would be cautious about describing this exercise as isolating an ESG effect, as the authors suggest in their response. The analysis provides evidence of robustness to the choice of ESG index, but it does not identify whether ESG characteristics play a role in explaining the observed relationships. I would therefore suggest clarifying this distinction and adjusting the interpretation accordingly.

Reviewer #2: The authors have adequately addressed the comments from the previous review. The revisions have improved the clarity of the manuscript, strengthened the robustness analyses, and clarified the interpretation of the results. In my opinion, the manuscript is scientifically sound and suitable for publication in PLOS ONE. I have no further comments and recommend acceptance.

**********

what does this mean?). If published, this will include your full peer review and any attached files.

If you choose “no”, your identity will remain anonymous but your review may still be made public.

**Do you want your identity to be public for this peer review?**  For information about this choice, including consent withdrawal, please see our Privacy Policy

Reviewer #1: No

Reviewer #2: No

**********

# Editor report: Acceptance letter

**DOI:** 10.1371/journal.pone.0341348.r004

**Authors:** Iván Barreda-Tarrazona

PONE-D-26-00622R1

PLOS One

Dear Dr. Cayon-Fallon,

I'm pleased to inform you that your manuscript has been deemed suitable for publication in PLOS One. Congratulations! Your manuscript is now being handed over to our production team.

At this stage, our production department will prepare your paper for publication. This includes ensuring the following:

* All references, tables, and figures are properly cited

* All relevant supporting information is included in the manuscript submission,

* There are no issues that prevent the paper from being properly typeset

You will receive further instructions from the production team, including instructions on how to review your proof when it is ready. Please keep in mind that we are working through a large volume of accepted articles, so please give us a few days to review your paper and let you know the next and final steps.

Lastly, if your institution or institutions have a press office, please let them know about your upcoming paper now to help maximize its impact. If they'll be preparing press materials, please inform our press team within the next 48 hours. Your manuscript will remain under strict press embargo until 2 pm Eastern Time on the date of publication. For more information, please contact onepress@plos.org.

You will receive an invoice from PLOS for your publication fee after your manuscript has reached the completed accept phase. If you receive an email requesting payment before acceptance or for any other service, this may be a phishing scheme. Learn how to identify phishing emails and protect your accounts at https://explore.plos.org/phishing.

If we can help with anything else, please email us at customercare@plos.org.

Thank you for submitting your work to PLOS One and supporting open access.

Kind regards,

PLOS One Editorial Office Staff

on behalf of

Dr. Iván Barreda-Tarrazona

Academic Editor

PLOS One
